# Acquired ichthyosis as a messenger to gastric diffuse large B-cell lymphoma^☆^

**DOI:** 10.1016/j.abd.2023.08.020

**Published:** 2024-07-14

**Authors:** İrfan Yavaşoğlu, Atakan Turgutkaya, Canten Tataroğlu, Ali Zahit Bolaman

**Affiliations:** a Division of Hematology, Aydin Adnan Menderes University Medical Faculty, Aydin, Turkey; b Division of Pathology, Aydin Adnan Menderes University Medical Faculty, Aydın, Turkey

*Dear Editor,*

A 50-year-old male patient presented with recurrent bloody vomiting. In the endoscopic examination, a tumoral lesion was detected in the gastric antrum. Immediate gastrectomy was performed due to perforation. Histopathological examination diagnosed a triple-expressor gastric Diffuse Large B-Cell Lymphoma (DLBCL) [Bcl-2 focal (60%), Bcl-6 focal (40%), C-Myc (10%), CD10 focal (70%)]. The Ki-67 index was positive at 60%. A stool examination of the Helicobacter pylori antigen was negative. PET-CT demonstrated no involvement at the operation (gastrectomy) site.

The patient had been complaining of itchy, polygon-shaped brown, gray, and white scales on the whole-body skin, more prominently on the extremities, for 2 years. At the same time, the skin was very dry and thickened (Fig. 1). There was no similar history in the patient’s family. The patient was started on R-CHOEP (Rituximab, Doxorubicin, Vincristine, Etoposide, Cyclophosphamide, and Prednisolone) chemotherapy. Also, a skin biopsy was performed. In the skin tissue hyperkeratosis, papillomatosis, mild acanthosis, and absent granular layer were noted. Perivascular mononuclear cell infiltration was observed in the superficial dermis (Fig. 2). At the end of chemotherapy, skin findings improved (Fig. 1).

**Figure 1:**
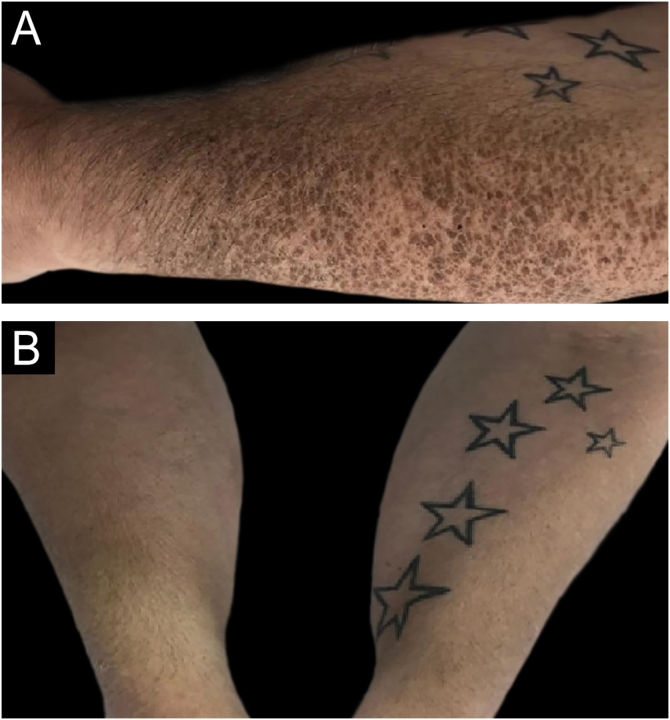
(A) Polygon-shaped brown, grey, or white scales on the forearm. (B) Resolution after the treatment.

**Figure 2:**
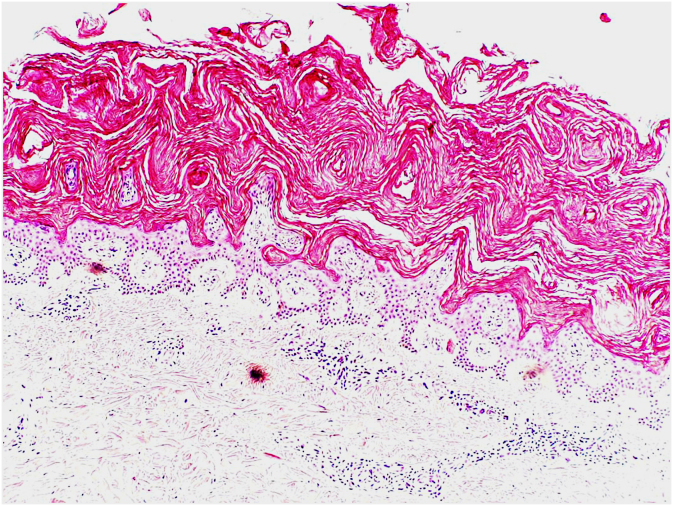
Orthohyperkeratosis, absence of granular cell layer and mild acanthosis are observed in the epidermis. In the upper dermis is a mild perivascular lymphocytic infiltrate (Hematoxylin & eosin, ×200).

Ichthyosis presents as rough, dry, skin with a large plate-like scale, and it can either be hereditary or acquired. Acquired Ichthyosis is defined in neoplastic disorders (Hodgkin's lymphoma, anaplastic large cell lymphoma, multiple myeloma, mycosis fungoides, POEMS [polyneuropathy, organomegaly, endocrinopathy, monoclonal protein, skin changes] syndrome, Kaposi's sarcoma, leiomyosarcoma, etc). Also, it is known to be associated with malnutrition, infections (HIV, Human T-lymphotropic virus), hypothyroidism, celiac disease, autoimmune conditions, sarcoidosis, graft-versus-host disease, and drug intake (hydroxyurea, allopurinol, vemurafenib, cholesterol-lowering medications, etc.). It is assumed that ichthyosis can be caused by impaired epidermal lipogenesis and production of transforming growth factor-α by tumor cells and impaired vitamin A metabolism.^1^, ^2^ According to the best of our knowledge, our case is the first ichthyosis as a precursor to gastric DLBCL.

## Financial support

None declared.

## Authors’ contributions

İrfan Yavaşoğlu: Design and planning of the study; Data collection, or analysis and interpretation of data; Drafting and editing of the manuscript or critical review of important intellectual content; Collection, analysis, and interpretation of data; Critical review of the literature; Approval of the final version of the manuscript.

Atakan Turgutkaya: Data collection, or analysis and interpretation of data; Drafting and editing of the manuscript or critical review of important intellectual content; Collection, analysis, and interpretation of data; Critical review of the literature.

Canten Tataroğlu: Data collection, analysis, and interpretation of data; Drafting and editing of the manuscript or critical review of important intellectual content.

Ali Zahit Bolaman: Design and planning of the study; Data collection, or analysis and interpretation of data; Drafting and editing of the manuscript or critical review of important intellectual content; Collection, analysis, and interpretation of data; Critical review of the literature; Approval of the final version of the manuscript.

## Conflicts of interest

None declared.
